# Editorial: Innovative methods in social robot behavior generation

**DOI:** 10.3389/frobt.2025.1741968

**Published:** 2025-12-01

**Authors:** Hifza Javed, Jauwairia Nasir, Antonio Andriella, WonHyong Lee, Mohamed Chetouani

**Affiliations:** 1 Honda Research Institute USA, San Jose, CA, United States; 2 University of Augsburg, Augsburg, Germany; 3 Institut de Robòtica i Informàtica Industrial (CSIC-UPC), Barcelona, Spain; 4 Handong Global University, Pohang, Republic of Korea; 5 Sorbonne Universités, Paris, France

**Keywords:** social robots, behavior generation, generative models, multimodal perception, large language models

## 1 Introduction

As social robots become increasingly embedded in our homes, workplaces, and educational settings, the demand for more intuitive, adaptive, and socially aware behavior generation has never been greater. Traditional approaches to robot behavior generation, such as Wizard-of-Oz and rule-based methods, have proven insufficient for achieving the fluidity and nuance that are required to meet the needs of human social interaction. The next-generation of social robots have to move beyond scripted exchanges to dynamically generate, adapt, and personalize their behaviors in real time, responding to multimodal cues and evolving social contexts.

This Research Topic “*Innovative Methods in Social Robot Behavior Generation*” was conceived to address this challenge. It brings together contributions that push the boundaries of how social robots perceive, plan, and act in ways that are meaningful, relevant, and “natural”. The selected works span technical innovations, empirical field studies, and a systematic analysis towards the shared goal of creating autonomous robotic systems capable of engaging humans through meaningful and contextually appropriate behaviors.

The five articles in this Research Topic collectively advance three core themes.

1. Generative Modeling Approaches: the integration of generative models, including LLMs, to enable open-ended, adaptive, and socially coherent robot interactions.
2. User Perception of Robot Behaviors: the investigation of how users perceive, interpret, and evaluate robot behaviors across diverse social and contextual settings, and
3. Autonomy in Social Robots: the development of autonomous systems that support ethical, context-aware social engagement.

## 2 Generative modeling approaches

As large language models (LLMs) become increasingly integrated into social robots, understanding how users respond to them over repeated interactions is crucial. Mauliana et al. explore this, in their paper *Exploring LLM-Powered Multi-Session Human-Robot Interactions with University Students*, through multi-session engagements between university students and EMAH, an LLM-powered humanoid robot designed for open-domain conversation. Across 4 weeks, the study found that while robot’s sociability, agency, and engagement remained stable, users perceived the robot as more lifelike over time without reduced discomfort. Despite occasional technical issues, sustained engagement highlighted the promise of compact, locally deployed LLMs for long-term interaction. The work underscores the need for improved robustness, personalization, and expectation management to support lasting social relationships with robots.

Additionally, Galatolo and Winkle propose an efficient multimodal behavior generation framework that addresses computational and ethical challenges in deploying social robots in low-resource or privacy-sensitive environments. Their work, *Simultaneous Text and Gesture Generation for Social Robots with Small Language Models*, introduces “gesture heads”—lightweight neural modules appended to the language model that operate in parallel with its text generation head to predict communicative intents, abstract representations of non-verbal behavior such as gestures or facial expressions, which can be mapped to platform-specific expressions. Comparing multiple strategies including In-Context Learning, Chain-of-Thought reasoning, and their proposed Gesture Head across LLaMA 3 models from 1B to 70B parameters, the authors show that their approach achieves strong accuracy and efficiency while maintaining real-time feasibility on Furhat and Pepper robots. The study advances the technical frontier of simultaneous text–gesture generation and highlights how small, open models can enable sustainable, ethical, and contextually coherent social robot interaction.

Finally, Shen and Johal present a culturally grounded diffusion-based framework for co-speech gesture generation that advances the field beyond culturally “neutral” gesture models. Their work, *TED-Culture: Culturally Inclusive Co-Speech Gesture Generation for Embodied Social Agents*, introduces the TED-Culture Dataset, a multilingual corpus spanning six languages (Indonesian, Japanese, German, Italian, French, and Turkish) with 17.5 h of 3D pose data from 183 speakers, specifically designed to capture culturally shaped gesture patterns. Building on this dataset, the authors develop DiffCulture, a Stable Diffusion–inspired generative model that maps speech audio and initial body poses to culturally coherent gesture sequences. The model achieves state-of-the-art performance on both the TED-Expressive and TED-Culture benchmarks, and is further validated on a NAO humanoid robot. This work demonstrates that culturally attuned gesture generation is both technically feasible and represents a key step toward more inclusive generative AI.

## 3 User perception of robot behaviors

In their paper *A field study to explore user experiences with Socially Assistive Robots (SARs) for older adults: emphasizing the need for more interactivity and personalisation*, Hofstede et al. present a comprehensive, multi-country field study examining how older adults, formal caregivers, and informal caregivers experience and evaluate Socially Assistive Robots in real home settings. Building on the Guardian project, the authors deployed the Misty II and Lizz robots, connected to caregiver and senior apps in households across Italy, Switzerland, and the Netherlands to explore how design factors such as personalization, interactivity, embodiment, and ethical considerations shape user experience. Through thematic analysis of interviews with 90 participants, six interrelated design factors emerged: personalization, interactivity, embodiment, ethical considerations, connectedness, and dignity. Personalization and interactivity were identified as the most influential, underscoring that end-users value adaptive communication, natural responsiveness, and contextual relevance over novelty or complexity. The findings highlight how human-centered, field-based evaluation can reveal social, ethical, and emotional dynamics often overlooked in laboratory research. This work situates user perception as a cornerstone for designing socially intelligent robots, demonstrating that meaningful, acceptable, and sustainable robot behavior generation must emerge from authentic engagement with users’ lived experiences.

## 4 Autonomy in social robots

In *Autonomy in Socially Assistive Robotics: A Systematic Review*, Maure and Bruno provide a systematic, PRISMA-based review of the past 5 years of Socially Assistive Robotics research, focusing on how varying levels of autonomy are achieved, communicated, and evaluated in human-robot interaction. Analyzing 70 studies across major HRI venues, they map the technological and methodological trends defining autonomous SARs, detailing how sensing, reasoning, and actuation capabilities are distributed between human operators and robotic systems. Their results reveal a dominance of short-term, dyadic studies using humanoid robots with partial autonomy, and highlight persistent gaps in multimodal perception, speech understanding, and transparency regarding autonomous functions. The review argues that autonomy in SARs is not merely a technical goal but a social and ethical challenge. By proposing the concept of Physically and Socially Assistive Robots, i.e., robots that fully leverage their embodiment, as well as social intelligence, the authors outline a path toward systems capable of adaptive, context-aware assistance in real-world environments, reinforcing autonomy as a cornerstone of next-generation social robot behavior generation.

## 5 Conclusion

This Research Topic demonstrates that advancing beyond scripted or rule-based paradigms requires both technical innovation and human-centered insight. The contributions collectively show how generative models, multimodal perception, and culturally grounded frameworks can enable social robots to act in ways that are adaptive, expressive, and ethically aligned. By combining novel behavior generation methods with sustained user engagement and systematic analysis of autonomy, these works lay the groundwork for more intuitive and contextually aware robotic systems that can truly participate in human social life.

Yet, important challenges remain. Ensuring robustness in real-world settings, maintaining transparency in generative processes, and achieving inclusivity across diverse users and cultures are essential next steps. Future research should deepen the integration of perception, generation, and ethics while establishing shared evaluation standards.

